# A Novel Approach by Integrating CT-Based Imaging Data and Machine Learning to Predict Patient-Specific Young's Modulus Values

**DOI:** 10.1155/aort/6257188

**Published:** 2025-08-14

**Authors:** Resmi S. L., Hashim V., Jesna Mohammed, Dileep P. N.

**Affiliations:** Department of Mechanical Engineering, TKM College of Engineering, Kollam, Kerala, India

**Keywords:** artificial neural network, bone mechanical properties, computed tomography, texture properties

## Abstract

Finite element analysis (FEA) stands as a cornerstone in preclinical investigations for implant therapy, particularly in orthopaedics and biomechanics. Accurate modelling of bone properties is crucial for meaningful FEA outcomes, considering the complex nature of bone tissue. This study proposes a novel approach by integrating CT-based imaging data and machine learning to predict patient-specific Young's modulus values. A back propagation neural network (BPNN), incorporating texture properties extracted from CT images, demonstrates robustness in predicting Young's modulus. Validation against three-point bending experiments on rabbit femur bones shows promising results, with stress values within 13% of those from FEA. The proposed methodology holds the potential for enhancing preclinical evaluations of implant therapy and fostering the development of patient-specific implants for improved clinical outcomes.

## 1. Introduction

Osteoporosis is a major global health issue, characterized by reduced bone mass and microarchitectural deterioration, which increases the risk of fragility fractures [1]. With an estimated global prevalence of 23.1% among women and 11.7% among men, osteoporosis significantly impairs quality of life and contributes to substantial morbidity, particularly in the elderly population [2]. The incidence of hip fractures, a severe consequence of osteoporosis, is projected to increase by 240% in women and 310% in men by 2050 [2].

Bone strength is a function of both bone density and mechanical properties such as stiffness, which is quantified using Young's modulus. This parameter plays a crucial role in evaluating bone quality and predicting fracture risk, especially in trabecular bone—an intricate, porous structure that varies in architecture and composition across multiple hierarchical levels [3, 4]. While macroscopic indicators like bone volume (BV) fraction (BV/trabecular volume (TV)) and anisotropy provide insight into structural integrity [5], the mechanical behaviour of bone is fundamentally governed by the intrinsic properties of the bone at the microscale [6].

Conventional methods for estimating Young's modulus include mechanical testing and empirical relationships based on CT-derived Hounsfield units (HU), but these approaches are limited by their invasiveness, generalization across populations and inability to capture patient-specific variability [7]. With the advent of machine learning, there is growing potential to model the complex relationships between CT imaging features and mechanical properties in a noninvasive, individualized manner.

Recent advancements in computational tools, like finite element analysis (FEA), have the potential to significantly enhance surgical outcomes by examining patient-specific three-dimensional models and their biomechanics [8]. Accurate values for Young's modulus are necessary for creating reliable biomechanical models and simulations of the skeletal system. These models are vital in researching how different forces impact bones and predicting the outcomes of surgeries or the performance of medical devices. These analyses play a crucial role in diagnosing and planning implant therapy, including aspects such as implant design, number of implants and placement sites [9]. A key challenge, however, lies in accurately estimating Young's modulus for bone structures from patient data.

Although bone mineral density (BMD) is considered to be the gold standard for the evaluation of bone strength and fracture risk, bone strength is determined by many other factors as bone microstructure and bone components. DXA is the most commonly used technique and offers benefits such as safety and ease of access; however, interpreting its results can be challenging, and positioning errors frequently occur in clinical practice. CT image provides information about the microarchitecture and microdamage of human cancellous bone specimens. Advanced imaging techniques like CT scans offer valuable insights into bone architecture, including parameters like bone volume fraction (BV/TV), trabecular thickness (Tb.T) and connectivity density (Conn.D.) [10]. Researchers have noted the importance of integrating both density and architecture to advance bone strength predictability to 90%–95% [11–13]. Hence, predicting bone strength requires an integrative approach that combines multiple factors beyond BMD, such as bone microstructure and mechanical properties. However, interpreting these complex structural data for bone strength prediction remains a challenge.

Texture analysis of CT images, particularly using methods like the grey-level co-occurrence matrix (GLCM), can provide valuable insights into bone microarchitecture and mechanical properties [6, 14, 15]. The GLCM quantitatively measures the frequency of different combinations of pixel brightness values occurring in an image. This method has shown promise in assessing trabecular bone microarchitecture, with 14 textural features derived from GLCM exhibiting a good correlation with bone mechanical properties [16]. Among these features, angular second moment (energy), contrast, correlation, entropy and the inverse difference moment (homogeneity) shows strong correlation with bone strength [16, 17]. Thus, the textural features offer a detailed representation of bone microarchitecture, enabling a more comprehensive assessment of bone strength.

Artificial neural networks (ANN) have become highly effective tools for data analysis and prediction across various disciplines. By simulating the human brain, ANN systems can identify patterns in complex datasets and forecast results. These models consist of an input layer, an output layer and one or more hidden layers with processing units known as neurons, allowing them to tackle nonlinear problems effectively [18, 19]. The backpropagation algorithm, a widely used training method for ANNs, minimizes the error function in weight space using gradient descent. This adaptive learning ability makes ANNs a reliable and powerful tool for data analysis and predictive modelling [20–23].

This study proposes a novel framework that integrates CT-based imaging data with machine learning algorithms to predict patient-specific Young's modulus values. By leveraging the detailed structural data available from CT scans and the pattern recognition capabilities of machine learning, this approach aims to provide a more accurate, scalable and clinically useful tool for assessing bone mechanical properties. Such advancements hold promise for improving osteoporosis diagnosis, enhancing personalized treatment planning and guiding implant design with greater biomechanical precision.

## 2. Methods

### 2.1. Data Acquisition

**Figure 1:**
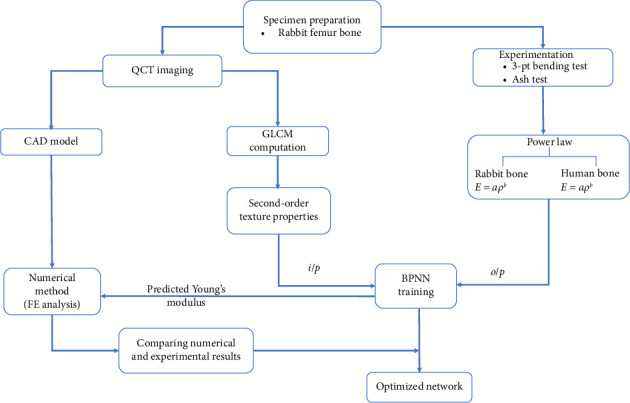
Overview of proposed work in predicting bone strength from CT image.

Figure 1 displays the outline of the proposed work in predicting bone strength from CT image.

**Figure 2:**
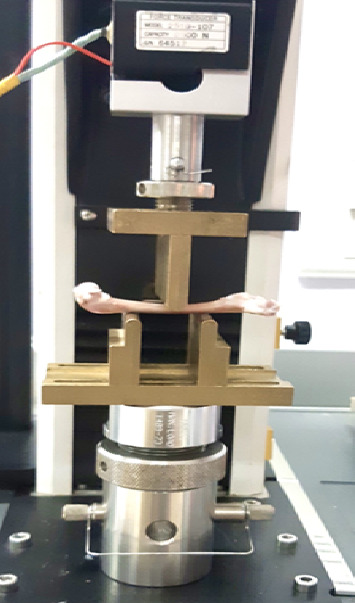
Three-point bending test setup with rabbit femur bone specimen.

Femur bone specimens of adult rabbit weighing approximately 1.75–2.5 kg were used for this study. Published methods for storage and handling of bone specimens were strictly followed during this investigation [24]. The specimens were stored at a temperature of −20°C in a saline solution. Subsequently, quantitative CT scans were conducted on specimens that were thawed at room temperature and maintained in a moist state using a sponge soaked in saline. After imaging, a three-point bending test was conducted on table-top UTM, INSTRON 3345, with Software BLUE HILL-3 at Sree Chitra Tirunal Institute for Medical Sciences and Technology, Kerala, India. Each specimen was placed on the jig as shown in Figure 2, and gauge length was set to 30 mm. Knife-edge indenter of 4-mm in tip radius was used within the three-point bending test. Elastic bending test was carried out at a crosshead speed of 5 mm/min at 24°C. Ash test was conducted after three-point bending test to determine the specimen's ash density. The bones were dried for the ash test at 110°C for an hour, then weighed (*w*1) to determine the dry weight. To obtain the ash, it is burned for a further 4 h at 600°C. Ash weight (*w*2) was observed, and ash density values are computed using the relation

(1) $$\begin{array}{c} \text{ash density}=\frac{\left(w1-w2\right)}{v1}. \end{array}$$

Initially the bone volume (*v*1) (mm^3^) is noted by water-displacement method. Ethics approval was obtained from the University ethics committee IAEC 1-KU-14/2019-20-TKM-PND (1).

**Figure 3:**
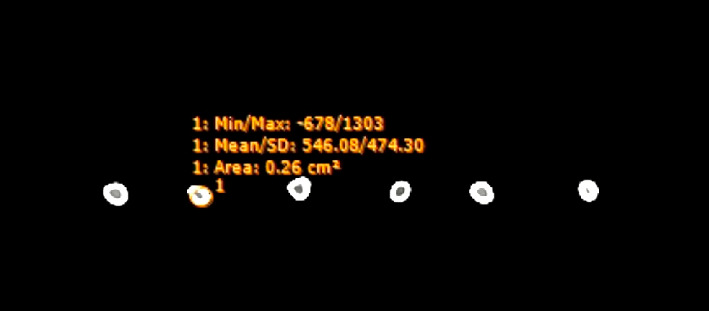
2D image with mean/SD of CT value.

The neural network is trained using a dataset obtained from QCT scanning (120 kV–347 mA, 0.625-mm slice thickness) of 15 rabbit femur bone specimens prepared as per the procedure reported [25]. A calibration phantom (Catphan) was laid on the CT scanner table and under the specimen. The training data matrix is further populated with QCT image property data of human skulls provided by a hospital in Kerala, India. The dataset used for testing the trained neural network is extracted from the clinical CT (120 kV–347 mA, 1-mm slice thickness) images of 15 rabbit femur bone of same specimen. The region of interest (ROI) or bone tissue considered for the study is limited to the bone range HU value to 250–1000. From each bone specimen, 10 equally spaced cross-sectional images from 5% to 95% of its length were selected to calculate HU values and texture property (GLCM) computation.  Figure 3 shows a 2D image with mean/SD of CT value or [HU] for the selected ROI.

### 2.2. Synthesis of GLCM and Second-Order Texture Properties From CT Image

The second-order texture parameters, such as energy, entropy, contrast, homogeneity and correlation, which are extracted from GLCM along with HU value, are used as the input values of the ANN. These parameters quantify the spatial relationship between pixels in the DICOM image and are extracted using MATLAB. The GLCM values with orientations 0°, 45° and 90° for a displacement of one unit for each selected ROI unit were extracted from the QCT images of rabbit bone and human specimens. The texture parameters are computed from average GLCM values using MATLAB code. Second-order texture metrics such as energy, entropy, contrast, homogeneity and correlation are extracted from GLCM, using MATLAB code. The parameter energy (*En*) measures the number of repeated pairs in the image. It is computed as the sum of squares of entries in the GLCM and is given by

(2) $$\begin{array}{c} En=\overset{Ng-1}{\underset{i=0}{\sum }}\overset{Ng-1}{\underset{j=0}{\sum }}P_{ij}^{2}; \end{array}$$

where *i* and *j* are the spatial coordinates of the function *P* (*i*, *j*) and *Ng* is the grey tone of the image.

The parameter entropy (*Et*) measures the randomness of grey level distribution within the image and is given by

(3) $$\begin{array}{c} Et=\overset{Ng-1}{\underset{i=0}{\sum }}\overset{Ng-1}{\underset{j=0}{\sum }}{-P}_{ij}*\log\;P_{ij}. \end{array}$$

The parameter contrast (*Co*) measures the local variations present in an image and is defined as

(4) $$\begin{array}{c} Co=\overset{N-1}{\underset{i=0}{\sum }}\overset{N-1}{\underset{j=0}{\sum }}{\left(i-j\right)}^{2}P\left(i,j\right). \end{array}$$

The parameter homogeneity (*Ho*) measures the uniformity of image and is defined as

(5) $$\begin{array}{c} Ho=\frac{\sum_{i=0}^{Ng-1}\sum_{j=0}^{Ng-1}P_{ij}}{1+{\left(i-j\right)}^{2}}. \end{array}$$

The parameter correlation (*C*) property measures the linear dependency of grey levels of neighbouring pixels and is given as

(6) $$\begin{array}{c} C=\frac{\sum_{i=0}^{N_{g-1}}\sum_{j=0}^{N_{g-1}}\left(I,j\right)p\left(i,j\right)-\mu_{x}\mu_{y}}{\sigma_{x}\sigma_{y}}, \end{array}$$

where *μ*_*x*_*μ*_*y*_ and*σ*_*x*_*σ*_*y*_ are the mean grey tone values and standard deviation (SD) of grey tone values, respectively.

Those computed texture values along with mean HU value from ROI are the input data set to ANN.

### 2.3. Computation of Modulus of Elasticity (*E*) of Rabbit and Human Bone

Young's modulus (*E*) was computed from three-point bending test of 15 rabbit femur bone samples after imaging using equation (7).

(7) $$\begin{array}{c} E=\left(\frac{F}{\Delta }\right)\cdot \frac{L^{3}}{48\cdot I}. \end{array}$$

 
(*F*/Δ) = slope of load -displacement graph
 
*L* = the span or the distance between supports
 
*I* = moment of inertia of the least cross section with respect to the *y* axis
 
*E* = the flexural elastic modulus for isotropic material

According to statistical analysis, the power law relationship connects apparent ash density (*ρ*) and Young's modulus (*E*) was formulated as *E* = 2.098247 ^∗^ *ρ*^0.126871^, with a standard error as 0.1383. Power law connects Young's modulus of human bone and density as established by Snyder and Schneider [26] and Helgason et al. [27] as *E* = 3.891 ^∗^ *ρ*^2.39^ [28]. The modulus of elasticity of both human and animal bone for training dataset was computed from the defined power laws.

### 2.4. Methods for Machine Learning-ANN Model

**Figure 4:**
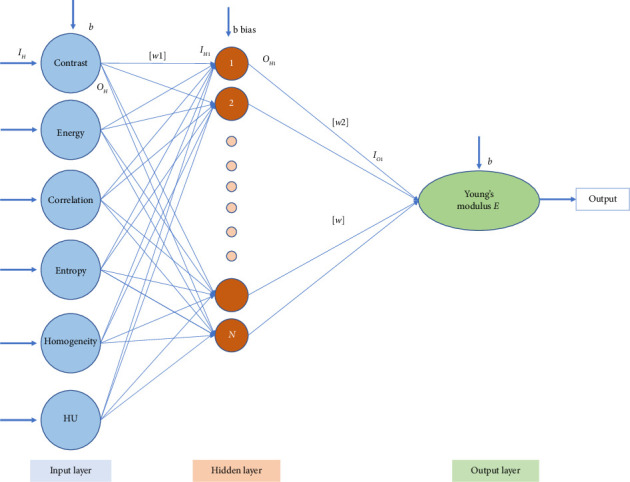
Schematic view of the neural network architecture developed.

Figure 4 shows graphic view of the neural network architecture developed. The neural network being proposed comprises three layers: an input layer with six parameters, a hidden layer and an output layer with a single value. Five texture properties such as energy, contrast, correlation, entropy and homogeneity and mean HU value from selected ROI (i.e., *i* = 6) served as inputs. The hidden layer consists of N neurons. A parametric study was carried out to identify a suitable value of N. The output layer has one neuron and the normalized Young's modulus (*E*). Tan-sigmoidal activation was considered for input and output neurons. The connecting weights between the input and hidden layers and the hidden and output layers are [*W*1] and [*W*2]. The values of [*W*1] and [*W*2] ranged from −1.0 to 1.0, while the bias value *b* was set between 0 and 1. MATLAB vR2011b (MathWorks Inc., Natick, MA) was used to model the neural network. The quality and quantity of training data affect the performance of a back propagation neural network (BPNN). Other factors include architecture, momentum constant and learning rate. [29–31]. In order to determine an optimal set of the above parameters, a study was carried out by varying one parameter at a time while keeping the other parameters constant.

The network used 300 sets of five texture features and their corresponding HU values extracted from QCT images of human and rabbit femur bone as input data set. The output target values, Young's modulus (*E*), were computed using regression equation established from experimental data. During the training phase, the network used 70% of dataset for training, 15% of dataset for validation and the remaining for testing the network.

### 2.5. Statistical Analysis

Descriptive statistical analysis was used for CT image texture properties and CT number. The image characteristics were expressed as mean ± SD, mode, median and range values.

## 3. Results

### 3.1. Image Characteristics of the Study Population

The image characteristics of study population are shown in Table 1. The mean value of CT number (HU) is 487.56 ± 153, which shows the range of bone tissue considered for the study. The mean values of the second order texture properties such as contrast, correlation, homogeneity, energy and entropy are 4.29 ± 2.07, 0.57 ± 0.32, 0.76 ± 0.09, 0.76 ± 0.09 and 3.57 ± 0.79, respectively.

### 3.2. Parametric Study on ANN Model

The validity of a neural network can be assessed by evaluating the regression coefficients (*R*^2^). A regression analysis was conducted using the training parameters specified in Table 2 to assess the advancement of the five networks developed. Given the dynamic changes in initial conditions and the sampling mean squared error (MSE) during iterative training, variations can occur. The findings were synthesized and are presented in Table 3. Reducing the MSE will lead to improved results. The MSE represents the average of the squared differences between the desired target and the output at each iteration. A regression value of 1 indicates a strong correlation between the target and output, while 0 suggests a random correlation. The training procedure halts when the improvement in generalization ceases.

### 3.3. Performance Parameters of the Prediction Neural Network Model

**Figure 5:**
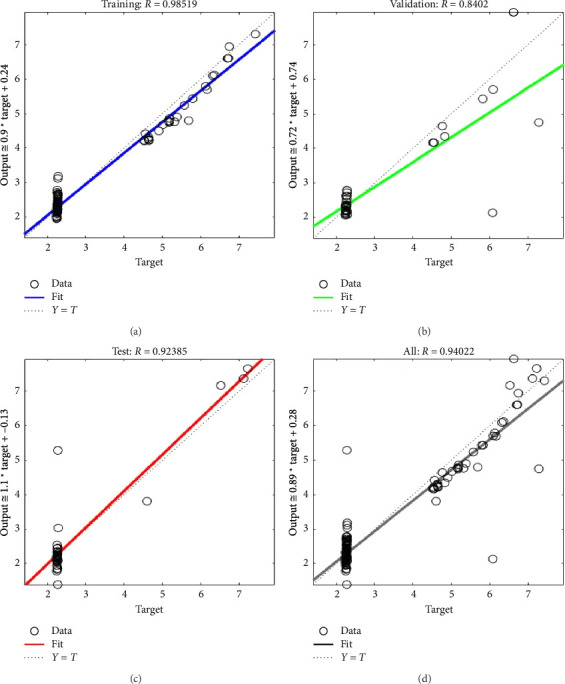
Comparison between Young's modulus from power law and ANN-predicted values for the (a) training dataset, (b) validation dataset, (c) test dataset and (d) overall performance.

The correlation coefficient (*R*) is used to quantify the strength of the linear relationship between the predicted output and the target output. This correlation coefficient is then plotted against the predicted and target Young's modulus values in Figures 5(a), 5(b), 5(c), 5(d).

**Figure 6:**
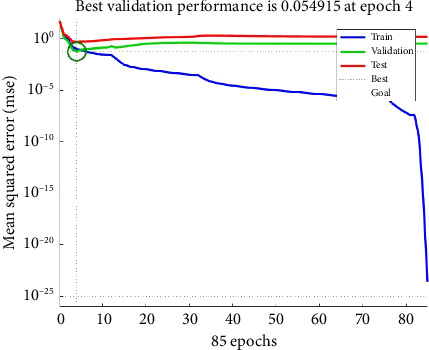
Validation performance graph between number of epochs and mean squared error.

It is noted from Figures 5(a), 5(b), 5(c) that the correlation coefficient between the target Young's modulus (*E*) and the neural network predicted Young's modulus for training, validating and testing data sets are 0.9851, 0.8402 and 0.9238, respectively. An overall correlation coefficient of 0.94022 has been obtained as shown in Figure 5(d), indicating a very good agreement with predicted Young's modulus and Young's modulus computed using power law during training phase which indicates convergence during ANN training. All curves in Figure 6 converges to a single point showing that the network performed equally well in training, validation and testing phases with an MSE of 0.054 at epoch 4.

### 3.4. Generation of Input Data Set for Training the Neural Network

The Young's modulus (*E*) and ash density values of 15 rabbit femur samples were computed from three-point bending test and ash test and are shown in Table 4. The power law is used for correlating the ash density and the Young's modulus values for generating input data set for training the neural network.

The correlation developed using power law between Young's modulus (*E*) and ash density (*ρ*) is given in equation (8) and was validated using the experimental values, with a standard error of 0.1383.

(8) $$\begin{array}{c} E=2.098247*\rho^{0.126871}. \end{array}$$

### 3.5. Performance Comparison of Prediction Model With Experimental Result

**Figure 7:**
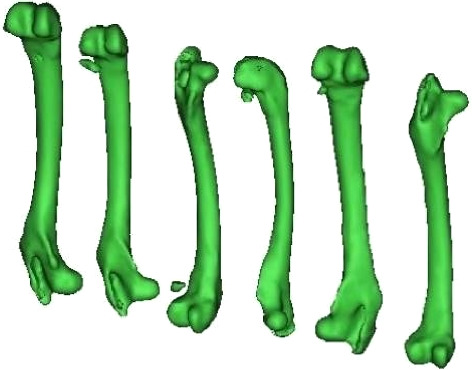
Rabbit femur bone model from CT image.

The accuracy of the trained neural network is further confirmed by comparing the maximum stress values obtained from experiments with those predicted through FEA of a rabbit femur bone model. The FEA is based on a model derived from DICOM images taken from CT scan data, which is processed using Materialise Mimics software.  Figure 7 depicts the rabbit femur model from CT image.

**Figure 8:**
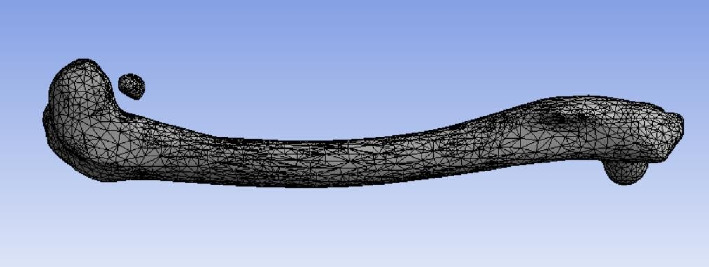
Meshed rabbit femur bone model.

The surface mesh for the femur bone model is generated after the model creation, in preparation for further FEA. A convergence technique is applied to refine the mesh size, ensuring an optimal mesh resolution. The mesh is composed of mixed elements, including tetrahedral and hexahedral, with triangular and quadrilateral faces at the boundaries.  Figure 8 illustrates the meshed model, which comprises 10,258 nodes and 39,290 elements.

**Figure 9:**
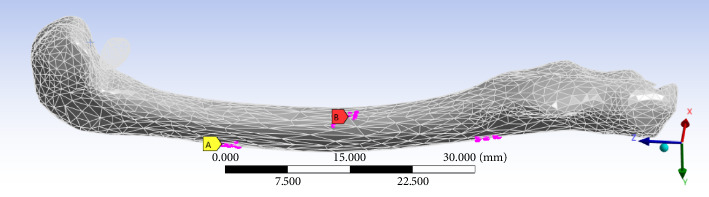
Rabbit femur bone model with loading and boundary condition.

Even though bone is heterogeneous and nonlinear, the current study characterizes material properties as homogeneous and linear in nature. The static structure module in ANSYS establishes boundary conditions for the femur bone model. A three-point bending span length of 30 mm was used, with a time-varying load applied at the midpoint.  Figure 9 shows the loading and boundary condition applied for the three-point bending analysis. The total deformation and equivalent stress were measured.

**Figure 10:**
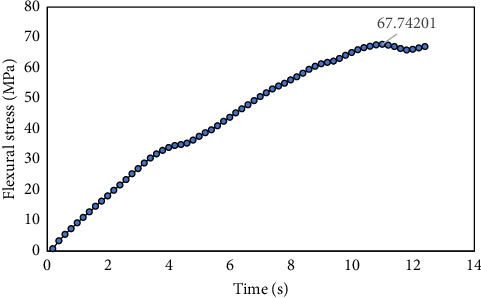
Flexural stress of rabbit femur bone from three-point bending test.

**Figure 11:**
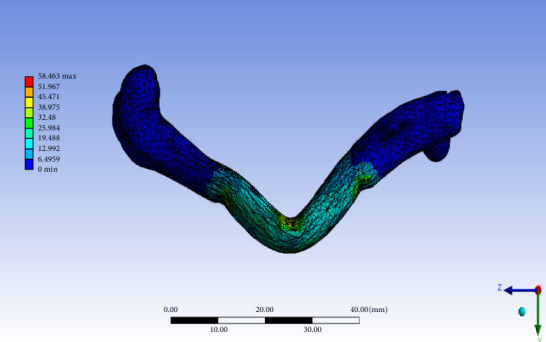
Contour plot from FEA of rabbit femur bone specimen.

The stress–time curve from the three-point bending test is presented in Figure 10 and shows a maximum flexural stress value of 67.74 MPa.  Figure 11 shows contour plot showing equivalent Von Mises stress from FEA using the model developed from the CT image of the experimental samples. The figure shows a maximum Von Mises stress value of 58.463 MPa.

## 4. Discussion

Since obtaining specimens for direct testing is challenging, the mechanical properties of patient-specific human bones, such as Young's modulus (*E*), are often predicted using the power law. However, this approach yields only approximate results because it does not consider bone quality. The machine learning algorithm proposed here overcomes this limitation by incorporating bone structure information along with BMD, which can be easily measured using DXA or QCT HU values, to more accurately predict bone strength.

Advancements in imaging technology have enabled the assessment of the structural integrity of both trabecular and cortical bone in three dimensions. Compared to existing techniques, the textural characteristics of microscopic or radiographic bone images provide localized information about the spatial arrangement of grey levels in a region, which describes bone architectural patterns. Second-order texture analysis investigates the relationship between architectural characteristics and bone mechanics by utilizing statistical metrics derived from the GLCM obtained from two-dimensional projection images. The CT value in the present study population ranged from 200 HU to 1200 HU, which ensures spongy bone tissue and cortical bone tissue in the samples. Texture characteristics such as contrast, homogeneity, correlation, energy and entropy were considered for the study due to their strong relationship with bone architecture and tissue mineralization. The ratio of the bone area in the image to the overall area under study is represented by the textural energy, which also indicates trabecular separation. The mean and SD in the study population (3.57 + 0.79; textural energy) show the range of trabecular separation, which indicates the bone features from cortical to cancellous bone.

Furthermore, studies have shown that BV/TV is the key factor influencing entropy and homogeneity [32]. As long as BV/TV remains static, homogeneity and entropy are textural characteristics that are independent of structure [33]. These variable values in the study population (range of entropy 5.18, homogeneity 0.47) show variation in the BV/TV of the sample population.

Data from a three-point bending and ash analysis test were used to create a power law relationship between Young's modulus and ash density. The standard error of the residual of the proposed power law relation quantifies the deviation of the predicted Young's modulus with the value obtained from the experiment. A value of 13.83% was obtained as the standard error for the predicted and experimental values of Young's modulus that is provided in Table 2. It may be noted that the data for specimen 5 has a significant residual value which might be owing to the slip-off of the specimen from the support during initial loading. The standard error calculated without considering the effect of the fifth specimen value is only 2.3%. The standard error improvement indicates the closeness of the predicted value with the experimental results.

The present study is novel method to explore the accuracy of machine learning on predicting patient-specific Young's modulus using the CT image properties. Young's modulus for each patient is predicted from six CT image properties using an optimal neural network with one hidden layer having 40 neurons. A population of 300 CT slices were used as the training, testing and validation data, which yields an accuracy of 0.98, 0.84 and 0.92, respectively. The overall regression coefficient (*R*^2^) of the proposed network was obtained as 0.94 that establishes its overall prediction accuracy.

The proposed network is then validated using FEA on a rabbit femur bone sample with the predicted Young's modulus. The maximum stress calculated by the FEA is 58.46 MPa. The bone model's assumption of isotropic linear behaviour accounts for the 13% deviation from the experimental results. The FEA value shows 13.83% less ultimate load than the experimental value of Young's modulus, which is safe in real-world scenarios. While numerical analysis provides approximations of real-life phenomena, the linear behaviour region strongly correlates with experimental results, making it ideal for implant selection and understanding the level of approximation relevant to practical applications.  Table 5 summarizes key studies related to the prediction of bone stiffness or Young's modulus using imaging and computational approaches, highlighting differences in methodology, validation and clinical relevance. The proposed study offers a novel, experimentally validated ANN-based approach that enables patient-specific predictions directly from CT data.

This study presents a novel, noninvasive approach to estimating patient-specific bone stiffness through the integration of CT-based imaging data and machine learning algorithms. The innovation lies in its ability to move beyond conventional population-based assessments and provide individualized predictions of Young's modulus, a key mechanical parameter that directly reflects bone strength and fracture susceptibility.

Unlike traditional methods that rely solely on HU correlations or mechanical testing—which are either too generalized or invasive—this framework captures complex microstructural features from high-resolution CT scans and uses machine learning to model their nonlinear relationship with mechanical properties. This allows for significantly enhanced prediction accuracy and personalization.

By focussing on the multiscale architecture of trabecular bone, the proposed method bridges the gap between image-derived structural parameters and their biomechanical implications at both tissue and organ levels. This is particularly valuable in clinical scenarios such as osteoporosis diagnosis, fracture risk assessment and orthopaedic implant design, where the mechanical interaction at the trabecular scale can determine treatment success.

Furthermore, the approach is fully compatible with existing imaging protocols, making it scalable, cost-effective and ready for integration into clinical workflows. Its application could significantly improve decision-making in bone health management, from early detection of osteoporosis to the customization of implants based on patient-specific mechanical profiles.

In conclusion, the microstructural characteristics and HU number from bone CT images are effectively utilized to predict the Young's modulus (*E*) of bone. Isotropic linear analysis, leveraging CT imaging and patient-specific Young's modulus, offers a valuable yet simplified method for understanding bone mechanics, with significant applications in clinical diagnostics, treatment planning and biomechanical research. By performing preclinical analysis using the network-predicted Young's modulus, clinicians can select the optimal implant dimensions and design. Furthermore, this approach contributes to the development of patient-specific implants that minimize the stiffness mismatch between bone and implant, ensuring a better fit, improved secondary stability and ultimately, a more favourable outcome for the patient.

## Figures and Tables

**Table 1:** Image characteristics of the study population.

Image properties	Mean ± SD	Mode	Median	Range	*p* value
Contrast	4.29 ± 2.07	6.33	4.5	10.23	*p* < 0.05
Correlation	0.57 ± 0.32	0.53	0.53	5.09	*p* < 0.008
Homogeneity	0.76 ± 0.09	0.75	0.76	0.47	*p* < 0.05
Energy	0.28 ± 0.12	0.35	0.26	0.75	*p* < 0.024
Entropy	3.57 ± 0.79	8.33	3.47	5.18	*p* < 0.05
CT number (HU value)	487.56 ± 153	276.52	471.08	655.93	*p* < 0.001

**Table 2:** Neural network training parameters.

Hidden layers	Epoch (iterations)	Time	Performance	Gradient	Validation check
10	106	0:00:00	8.5e − 05	0.0001736	100
20	111	0:00:00	8.8e − 05	0.000185	100
30	103	0:00:00	1.65e − 05	0.000145	100
40	85	0:00:00	2.45e − 24	1.9144e − 12	81
45	66	0:00:00	3.42e − 28	1.4881e − 14	62

**Table 3:** Neural network tuning parameters.

Hidden layers	Method	No. of samples	Mean square error	Regression coefficient
10	Training	210	0.31281	0.930
	Validation	45		0.90

20	Training	210	0.60903	0.909
	Validation	45		0.77831

30	Training	210	0.42041	0.9613
	Validation	45		0.8245

40	Training	210	0.054915	0.968
	Validation	45		0.94

45	Training	210	0.5323	0.97704
	Validation	45		0.85

**Table 4:** Calculated value of ash density and Young's modulus (*E*) from experimental data.

Bone sample	Ash density (× 10^−3^ kg/m^3^)	Young's modulus (*E*) (MPa)	Young's modulus (*E*) from power law (MPa)	Residual
1	0.74	2.4637	2.019603	0.197276
2	0.71	1.6840	2.009026	0.105642
3	0.5	1.7633	1.921608	0.025039
4	0.84	2.1920	2.052342	0.019527
5	0.63	0.5100	1.978786	2.15712
6	0.74	2.2376	2.019603	0.047562
7	0.42	2.3394	1.879568	0.211446
8	0.59	2.0377	1.962386	0.00568
9	0.84	2.4293	2.052342	0.142135
10	1.45	2.4059	2.199528	0.042598
11	1.97	2.6393	2.286734	0.124338
12	1.35	2.4084	2.179677	0.052356
13	1.25	2.6806	2.158498	0.272601
14	1.71	1.94991	2.246037	0.087691
15	1.5	2.80716	2.209008	0.357785

**Table 5:** Comparative overview of methods for predicting bone mechanical properties from imaging data.

Study	Method used	Input data	Output predicted	Is experimentally validated?	Is patient-specific?	Clinical utility
Rho et al. [34]	Empirical model	HU (CT scans)	Young's modulus (linear fit)	Partially	Generalized values	Limited (not accurate for individuals)
Chen et al. [35]	Linear regression	Micro-CT features	Apparent modulus	Yes	Average per region	Research focussed
Knowles et al. [36]	Analytical model	Density, anisotropy	Elastic constants	Yes	General assumptions	Research or offline analysis
Shirvaikar et al. [37]	ML model (SVM/random forest)	Bone morphometry	Modulus range	No	Slightly	Promising, but lacks physical validation
Current study (2025)	Artificial neural network (ANN)	CT-based structural features (e.g., BV/TV, HU or density, texture features)	Young's modulus	Yes – trained on real experimental data	Fully patient-specific	Non-invasive, real-time estimation, supports diagnosis and implant planning

## Data Availability

Data will be made available upon request.
